# Atypical Haemolytic Uraemic Syndrome Associated with* Clostridium difficile* Infection Successfully Treated with Eculizumab

**DOI:** 10.1155/2018/1759138

**Published:** 2018-05-10

**Authors:** Joshua M. Inglis, Jeffrey A. Barbara, Rajiv Juneja, Caroline Milton, George Passaris, Jordan Y. Z. Li

**Affiliations:** ^1^Department of General Medicine, Royal Adelaide Hospital, Adelaide, SA, Australia; ^2^Department of Renal Medicine, Flinders Medical Centre, Adelaide, SA, Australia; ^3^School of Medicine, Flinders University, Adelaide, SA, Australia

## Abstract

*Clostridium difficile* infection is a rare precipitant of atypical haemolytic uraemic syndrome (aHUS). A 46-year-old man presented with watery diarrhoea following an ileocaecal resection. He developed an acute kidney injury with anaemia, thrombocytopaenia, raised lactate dehydrogenase, low haptoglobin, and red cell fragments. Stool sample was positive for* C. difficile *toxin B. He became dialysis-dependent as his renal function continued to worsen despite treatment with empiric antibiotics and plasma exchange. The ADAMTS13 level was normal consistent with a diagnosis of aHUS. The commencement of eculizumab led to the resolution of haemolysis and stabilisation of haemoglobin and platelets with an improvement in renal function.

## 1. Background

Atypical haemolytic uraemic syndrome (aHUS) is a rare disorder characterised by thrombocytopaenia and evidence of microangiopathic haemolysis. ADAMTS13 levels are normal and STEC (Shiga toxin-producing* E. coli*) is not present in the stool. The pathogenesis involves activation of complement via the alternative pathway leading to a thrombotic microangiopathy with end-organ involvement predominantly affecting the renal and neurological systems [1]. The precipitants for aHUS include complement regulation deficits, infections, drugs, and pregnancy [2].* Clostridium difficile* infection has been identified as a rare precipitant [3–7].

## 2. Case Report

A 46-year-old man with no past medical history and no regular medications presented to a rural hospital with an acute abdomen. An explorative laparotomy revealed a small bowel obstruction secondary to a congenital band with an associated strangulated segment that required an ileocaecal resection. The patient received antibiotic cover with cephazolin, gentamicin, and metronidazole in addition to opioid analgesia. On the tenth postoperative day he had watery diarrhoea. On the following day the patient became anuric with moderate hypertension (BP 160/80).

Blood tests revealed an acute kidney injury with a sudden rise in creatinine from 71 to 307 *μ*mol/L (reference range RR 50–120 *μ*mol/L). The blood film showed red cell fragments (18–24 per HPF) together with an acute anaemia, nadir haemoglobin 68 g/L (RR 110–150 g/L), and thrombocytopaenia, nadir platelet count 87 × 10^9^/L (RR 150–450 × 10^9^/L). Further blood tests revealed a lactate dehydrogenase (LDH) of 1657 (RR 110–230 U/L), a haptoglobin of 0.09 (RR 0.50–2.50), a bilirubin of 86 (RR 2–24), and a negative direct antibody test (DAT). Complement components were low with C3 of 0.75 (RR 0.85–1.60) and C4 of 0.09 (RR 0.12–0.36). There was a coinciding derangement in the liver function tests with gamma-glutamyl transferase (GGT) 589 IU/L (RR < 60 IU/L), alkaline phosphatase (ALP) 112 IU/L (RR < 55 IU/L), aspartate transaminase (AST) 60 IU/L (RR < 55 IU/L), and alanine transaminase (ALT) 209 IU/l (RR < 45 IU/L) which was attributed to critical illness. There was an associated coagulopathy with INR 4.9 (range 0.9–1.2), APTT 67 (range 24–38), fibrinogen 3.0 (range 1.5–4), and D-dimer 3.2 that had likely resulted from the hepatic dysfunction.

The stool sample was positive for* Clostridium difficile* toxin B and negative for Shiga toxin on two occasions. Stool culture was negative for other enteric pathogens. ADAMTS13 activity was 69% which is within the normal reference range. Autoimmune screen for ANA, dsDNA, and ANCA was negative. Renal tract ultrasound revealed normal sized kidneys with no hydronephrosis. Liver ultrasound showed hepatomegaly (18 cm) with no evidence of biliary obstruction. Complement factor H* (CFH)* gene analysis did not reveal a mutation.

The patient received daily plasma exchange and haemodialysis in the Intensive Care Unit. Intravenous metronidazole and oral vancomycin were given as antibiotic therapy for* C. difficile*. Multiple blood transfusions were required to maintain the haemoglobin. The patient required intubation and ventilation for hypoxic respiratory failure for a period of 18 days.

Eculizumab infusions were commenced with the cessation of plasma exchange. The protocol involved infusions of 900 mg weekly for four weeks followed by a single infusion of 1200 mg in the fifth week. During the maintenance phase the patient received fortnightly infusions of 1200 mg for one year.

Eculizumab treatment led to a normalisation in haemoglobin, platelet count, LDH, and haptoglobin over the following fortnight. The patient then became dialysis-independent and serum creatinine stabilised at 200 *μ*mol/L prior to discharge.

He continued to receive eculizumab as an outpatient for one year. There was no relapse of disease on eculizumab cessation. He continues to be monitored with regular blood tests. His renal function remains stable at a new baseline creatinine of 130 *μ*mol/L and there is no evidence of haemolysis.

## 3. Discussion

Atypical haemolytic uraemic syndrome is a clinical diagnosis based on microangiopathic haemolytic anaemia, thrombocytopaenia, and acute kidney injury in the absence of STEC with normal ADAMTS13 activity. Episodes may be precipitated by infection in patients with a genetic susceptibility to complement dysregulation. However, specific complement gene pathway mutations are found in only 50–60% of cases of true aHUS [8].

This case provides further evidence for* C. difficile* infection as a rare precipitant of aHUS. There are seven reported adult cases of* C. difficile*-associated aHUS with this being the first to occur in an adult male and following abdominal surgery [3–7]. The characteristics of these cases are listed in Table 1. While all cases have shared similar clinical presentations of anaemia, thrombocytopaenia and acute kidney injury, the nature of diarrhoea has varied with both watery and bloody stools being reported. Confusion has been reported in four of eight reported cases suggesting that neurological involvement may occur. However, confusion was not present in our case.

Various treatments have been used in the reported cases of* C. difficile*-associated aHUS including antibiotics, steroids, and plasma exchange with generally favourable outcomes in all but one patient who developed allograft failure and required graft nephrectomy [7]. There have been no documented recurrences of* C. difficile*-associated aHUS.

Terminal complement inhibitors have emerged as an effective therapy for aHUS. Eculizumab has been shown to control haemolysis and lead to improvements in renal function [9]. This is the first reported case of* C. difficile*-associated aHUS to be treated with eculizumab in addition to conventional therapies. The patient responded favourably with resolution of haemolysis, normalisation of haemoglobin and platelets, and an improvement in renal function. There has been no recurrence of disease two years after ceasing eculizumab. Eculizumab may be an effective agent for achieving resolution of haemolysis and stabilising renal function in patients with* C. difficile*-associated aHUS.

## Figures and Tables

**Table 1:** Published reports of *C. difficile*-associated aHUS.

Reference	Age/sex	Kidney	Clinical features	Treatment	Dialysis	Outcome
[3]	51/female	Native	Watery diarrhoea, confusion	Vancomycin	No	Recovery
[4]	46/female	Native	Bloody diarrhoea, vomiting	Metronidazole, plasmapheresis	Yes	Recovery
[5]	62/female	Native	Watery diarrhoea	Metronidazole, plasmapheresis, steroids	Yes	Recovery
[6]	73/female	Native	Watery diarrhoea, respiratory distress, chills, anuria	Metronidazole, steroids	Yes	Recovery
[7]	29/female	Transplant	Diarrhoea, decreased urine output	Vancomycin	Yes	Complete recovery
[7]	52/female	Transplant	Fever, diarrhoea, nausea, vomiting	Metronidazole, plasmapheresis	Yes	Allograft failure and transplant nephrectomy
[7]	63/female	Native	Bloody diarrhoea, fever, confusion	Metronidazole, vancomycin, plasmapheresis	Yes	Partial recovery
Current case	46/male	Native	Watery diarrhoea, anuria	Plasmapheresis, metronidazole, vancomycin and eculizumab	Yes	Recovery
